# NMDA Receptor Antagonists Degrade Lipofuscin via Autophagy in Human Retinal Pigment Epithelial Cells

**DOI:** 10.3390/medicina58081129

**Published:** 2022-08-20

**Authors:** Jae Rim Lee, Kwang Won Jeong

**Affiliations:** College of Pharmacy, Gachon Research Institute of Pharmaceutical Sciences, Gachon University, 191 Hambakmoero, Yeonsu-gu, Incheon 21936, Korea

**Keywords:** retinal pigment epithelial cells, drusen, *N*-retinylidene-*N*-retinylethanolamine (A2E), *N*-methyl-D-aspartate (NMDA), autophagy

## Abstract

*Background and Objectives:* Age-related macular degeneration is a slow-progressing disease in which lipofuscin accumulates in the retina, causing inflammation and apoptosis of retinal pigment epithelial (RPE) cells. This study aimed to identify *N*-methyl-D-aspartate (NMDA) signaling as a novel mechanism for scavenging *N*-retinylidene-*N*-retinylethanolamine (A2E), a component of ocular lipofuscin, in human RPE cells. *Materials and Methods:* A2E degradation assays were performed in ARPE-19 cells using fluorescently labeled A2E. The autophagic activity in ARPE-19 cells was measured upon blue light (BL) exposure, after A2E treatment. Autophagy flux was determined by measuring LC3-II formation using immunoblotting and confocal microscopy. To determine whether autophagy via the NMDA receptor is involved in A2E clearance, ATG5-deficient cells were used. *Results:* Ro 25-6981, an NR2B-selective NMDA receptor antagonist, effectively cleared A2E. Ro 25-6981 reduced A2E accumulation in the lysosomes of ARPE-19 cells at sub-cytotoxic concentrations, while increasing the formation of LC3-II and decreasing p62 protein levels in a concentration-dependent manner. The autophagic flux monitored by RFP-GFP-LC3 and bafilomycin A1 assays was significantly increased by Ro 25-6981. A2E clearance by Ro 25-6981 was abolished in ATG5-depleted ARPE-19 cells, suggesting that A2E degradation by Ro 25-6981 was mediated by autophagy. Furthermore, treatment with other NMDA receptor antagonists, CP-101,606 and AZD6765, showed similar effects on autophagy activation and A2E degradation in ARPE-19 cells. In contrast, glutamate, an NMDA receptor agonist, exhibited a contrasting effect, suggesting that both the activation of autophagy and the degradation of A2E by Ro 25-6981 in ARPE-19 cells occur through inhibition of the NMDA receptor pathway. *Conclusions:* This study demonstrates that NMDA receptor antagonists degrade lipofuscin via autophagy in human RPE cells and suggests that NMDA receptor antagonists could be promising new therapeutics for retinal degenerative diseases.

## 1. Introduction

Age-related macular degeneration (AMD) is the most common disease diagnosed in people over 50 years of age and is a leading cause of rapid vision loss [1,2]. There are two types of AMD: wet AMD (neovascular) and dry AMD (non-neovascular). In wet AMD, new blood vessels are formed from the choroid that grows into the retina, resulting in rapid vision loss [3]. In contrast, dry AMD is a slow-progressing disease in which drusen accumulate between the retinal pigment epithelial (RPE) cell layer and Bruch’s membrane. Currently, wet AMD is treated using vascular endothelial growth factor (VEGF) inhibitors [4,5]. However, the exact cause of dry AMD has not been elucidated, which has limited the development of treatments for dry AMD. Compounds that inhibit poly (ADP-ribose) polymerase-1 (PARP1) [6] and certain antioxidants such as lutein, zeaxanthin, and resveratrol [7,8,9] have been proposed for treating dry AMD. Drugs with antioxidant properties, complement cascade inhibitors, neuroprotective agents, visual cycle inhibitors, gene therapy, and cell-based therapies have also been reported as alternative approaches for the prevention and treatment of dry AMD [10]. However, none of these therapies have shown satisfactory efficacy in treating dry AMD [11].

Drusen, a pathological feature of dry AMD, contains lipofuscin, which are lipid deposits [12]. Bis-retinoid *N*-retinyl-*N*-retinylidene ethanolamine (A2E) is a component of lipofuscin that damages RPE cells [13,14]. A2E is a fluorescent substance formed during visual activity that consists of all-trans-retinal and ethanolamine in a 2:1 ratio. A2E accumulates in the lysosomes around the nucleus of RPE cells and is converted to A2E-epoxide upon exposure to blue light (BL), which generates reactive oxygen species [15]. The free radicals produced by photooxidation impair the function of RPE cells and interfere with the interaction between Bruch’s membrane and the RPE cell layer [16,17]. Furthermore, A2E induces inflammation and apoptosis in RPE cells [18,19,20]. Therefore, the removal of A2E from RPE cells could be beneficial for the prevention and treatment of dry AMD. A previous study showed that photooxidation of A2E by blue light affects a wide range of cellular physiological pathways, including TNFα signaling, hypoxia, the p53 pathway, IL2 STAT signaling, complement, and apoptosis in retinal pigment epithelial cells [21,22]. Therefore, the fundamental approach to overcome the effects of A2E photooxidation is to remove A2E, a phototoxic factor accumulated in cells in the process of aging, and a new cell physiology mechanism that enables the elimination of A2E needs to be discovered.

This study aimed to identify *N*-methyl-D-aspartate (NMDA) signaling as a novel mechanism for scavenging A2E, which could also be a novel therapeutic molecule for retinal degenerative diseases. For this purpose, compounds with the ability to clear A2E were screened from a library of compounds with known mechanisms of action. Ro 25-6981, an NR2B-selective NMDA receptor antagonist, was confirmed to be effective in eliminating A2E, thereby exhibiting the potential to treat retinal degenerative diseases. We also revealed that autophagy is involved in A2E clearance by Ro 25-6981. Taken together, our findings suggest that NMDA receptor antagonists degrade lipofuscin via autophagy in human RPE cells and might serve as useful tools for future research on retinal degenerative diseases

## 2. Materials and Methods

### 2.1. Cell Culture and Reagents

The human RPE cell line, ARPE-19, was cultured in Dulbecco’s modified Eagle medium, F-12 (DMEM F-12; WELGENE, Gyeongsan, Korea) containing 10% fetal bovine serum (FBS) at 37 °C in a humidified incubator with 5% CO_2_. Ro 25-6981 maleate salt, L-glutamic acid, AZD6765, and CP-101,606 were purchased from Sigma-Aldrich (St. Louis, MI, USA). A2E was purchased from Key Synthesis LLC (Philadelphia, PA, USA). Rapamycin was purchased from Cayman Chemical Co. (Ann Arbor, MI, USA). Bafilomycin A1 (Baf A1) was purchased from Abcam (Cambridge, UK).

### 2.2. A2E Degradation Assay

A2E degradation assay was performed in ARPE-19 cells using fluorescently labeled A2E (A2E-BDP) [23]. Briefly, ARPE-19 cells were treated with A2E-BDP (10 μM) for 24 h and then treated with a compound for 48 h. Fluorescence intensity was measured using a microplate reader.

### 2.3. Cell Viability Assay

ARPE-19 cells were seeded in 24-well plates at a density of 2 × 10^4^ cells per well and treated with Ro 25-6981 or glutamate at 37 °C for 5 days at specified concentrations. Cell viability was determined using an EZ-Cytox assay kit (DoGenBio, Seoul, Korea). The absorbance was measured at 450 nm using the BioTek microplate reader (Winooski, VT, USA).

### 2.4. Confocal Microscopy

ARPE-19 cells were treated with A2E (5 μM) three times at 48 h intervals. After A2E accumulation, the cells were treated with Ro 25-6981 twice at 24 h intervals and stained with LysoTracker Red DND-99 (Thermo Fisher, Rockford, IL, USA) and incubated in an incubator at 37 °C for 1 h. Nuclei were stained with 1 μg/mL of Hoechst 33342 (Thermo Scientific). The samples were visualized using a confocal fluorescence microscope (Nikon, Tokyo, Japan).

### 2.5. BL-Induced RPE Damage Model in ARPE-19 Cells

ARPE-19 cells were seeded in 6-well plates at a density of 2 × 10^4^ cells per well and treated thrice with A2E (5 μM) at 48 h intervals. Following this step, the cells were treated with Ro 25-6981 or glutamate twice at 24 h intervals and then exposed to BL (430 nm) for 30 min. Finally, the cells were incubated for further 24 h and cell lysates were prepared for western blotting.

### 2.6. RNA Interference and Transfection

ARPE-19 cells were transfected with small interfering RNA that targeted the autophagy-related 5 (*ATG5*) mRNA (siATG5) or non-specific siRNA (siNS). ARPE-19 cells were transfected using Oligofectamine (Invitrogen, Carlsbad, CA, USA) according to the manufacturer’s protocol. The sequences of the siRNAs were as follows: siNS, 5′-UUCUCCGAACGUGUCACGUTT-3′ (sense) and 5′-ACGUGACACGUUCGGAGAATT-3′ (antisense); siATG5, 5′-GAUUCAUGGUGAGCCAdTdT-3′ (sense) and 5′-UGGCUCAAUUCCAUGAAUCdTdT-3′ (antisense).

### 2.7. Western Blotting

ARPE-19 cell lysates were prepared using a RIPA buffer [50 mM Tris-HCl pH 8.0, 150 mM NaCl, 2 mM ethylenediaminetetraacetic acid, 1% sodium dodecyl sulfate (SDS), 1% sodium deoxycholate, and 1% NP-40]. Subsequently, SDS–polyacrylamide gel electrophoresis was performed, and the proteins were transferred to Immun-Blot polyvinylidene difluoride (PVDF) membranes (Bio-Rad, Hercules, CA, USA). The antibodies used were as follows: anti-p62, anti-phospho-p62 (S349), anti-LC3, anti-ATG5 (Cell Signaling Technology, Danvers, MA, USA), and β-actin (Santa Cruz Biotechnology Inc., Santa Cruz, Dallas, TX, USA). Images were quantified using the Image J software (version 1.8.0, National Institutes of Health, Bethesda, MD, USA).

### 2.8. Measurement of Autophagic Flux

ARPE-19 cells were cultured in 6-well plates at a density of 6 × 10^4^ cells per well and transiently transfected with mCherry-eGFP-LC3 plasmid expressing an RFP-GFP-LC3 construct using Lipofectamine™ 2000 (Invitrogen, Carlsbad, CA, USA), following the manufacturer’s instructions. After 72 h of transfection, the cells were treated with Ro 25-6981 for 8 h and then fixed with 4% formalin. Hoechst 33342 staining was performed to determine the degree of A2E accumulation and cellular apoptosis induced by BL irradiation, and the slides were examined using a laser scanning microscope (Nikon, Tokyo, Japan). Additionally, LC3-II levels were measured in samples treated with Baf A1, Ro 25-6981, or both compounds simultaneously for 8 h. Quantification of LC3-II band density was performed using ImageJ software.

### 2.9. Statistical Analysis

Statistical analyses were performed using Prism 8 software (GraphPad Inc., San Diego, CA, USA). Data are presented as the mean ± standard deviation, and significant differences among groups were calculated using one-way analysis of variance (ANOVA, parametric) followed by a Duncan’s test or *t*-test. Statistical significance was accepted at a *p* value < 0.05.

## 3. Results

### 3.1. Ro 25-6981 Reduces A2E in ARPE-19 Cells

**Figure 1:**
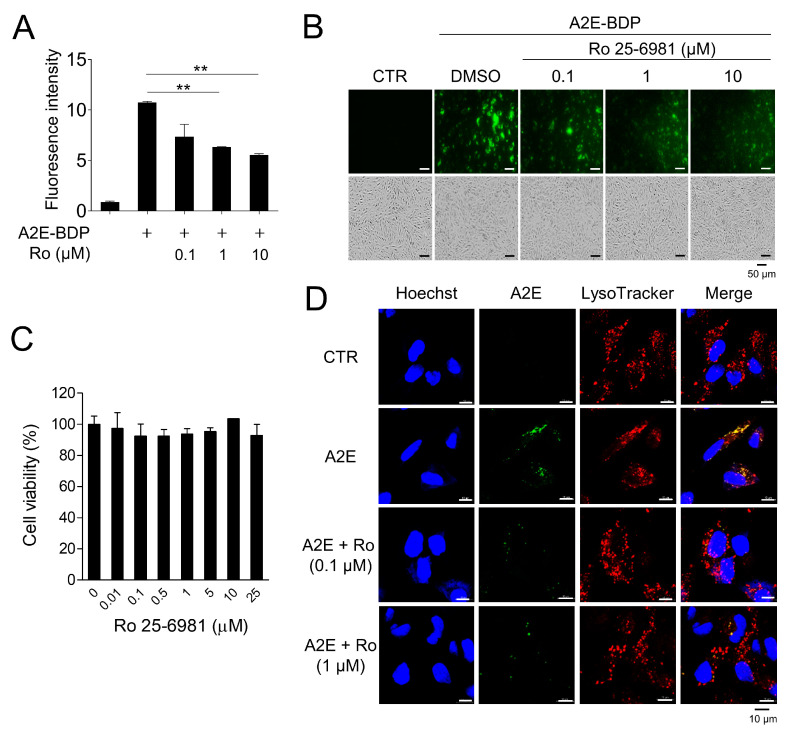
A2E degradation by Ro 25-6981 in ARPE-19 cells. (**A**) ARPE-19 cells with accumulated A2E-BDP (10 μM) for 24 h were treated with Ro 25-6981 (0.1 μM, 1 μM, and 10 μM) for 48 h. The ability of Ro 25-6981 (Ro) to clear A2E-BDP was confirmed using a microplate reader. (**B**) Ro 25-6981 clears intracellular A2E-BDP in a concentration-dependent manner. Intracellular A2E-BDP was observed using a fluorescence microscope. (**C**) Cell viability of Ro 25-6981 in ARPE-19 cells. ARPE-19 cells were treated with various concentrations of Ro 25-6981. After incubation, cell viability was calculated using an EZ-Cytox assay kit. (**D**) The effect of Ro 25-6981 (Ro) on A2E levels in ARPE-19 cells was monitored using fluorescence confocal microscopy. ARPE-19 cells with accumulated A2E (5 μM) were treated with Ro 25-6981 (0.1 or 1 μM) or vehicle (CTR). The fluorescence signals observed were for LysoTracker (red) and A2E (green). Representative results are presented as the mean ± S.D. (*n* = 3). ** *p* < 0.01 vs. A2E-BDP.

Through the screening of a chemical library (Tocriscreen Plus Micro, Tocris Bioscience, Bristol, UK), Ro 25-6981, a GluN2B-selective NMDA receptor antagonist, was identified as an effective compound for A2E removal. Ro 25-6981 cleared A2E-BDP that accumulated in ARPE-19 cells in a concentration-dependent manner (Figure 1A,B). Moreover, at the concentrations used in our experiment, Ro 25-6981 did not exhibit cytotoxicity (Figure 1C), suggesting that A2E clearance by Ro 25-6981 was not due to the release of A2E upon cell death. A2E accumulates in the lysosomes of RPE cells [24]. Therefore, the effect of Ro 25-6981 treatment on lysosomal A2E levels was observed using confocal fluorescence microscopy after staining with LysoTracker. As expected, A2E accumulated at high levels in the lysosomes of RPE cells. However, upon treatment with Ro 25-6981 (0.1 μM and 1 μM), the levels of lysosomal A2E were significantly reduced (Figure 1D). Taken together, the NMDA receptor antagonist Ro 25-6981 effectively reduces A2E accumulation in the lysosomes of RPE cells.

### 3.2. Activation of Autophagy by Ro 25-6981 in ARPE-19 Cells

**Figure 2:**
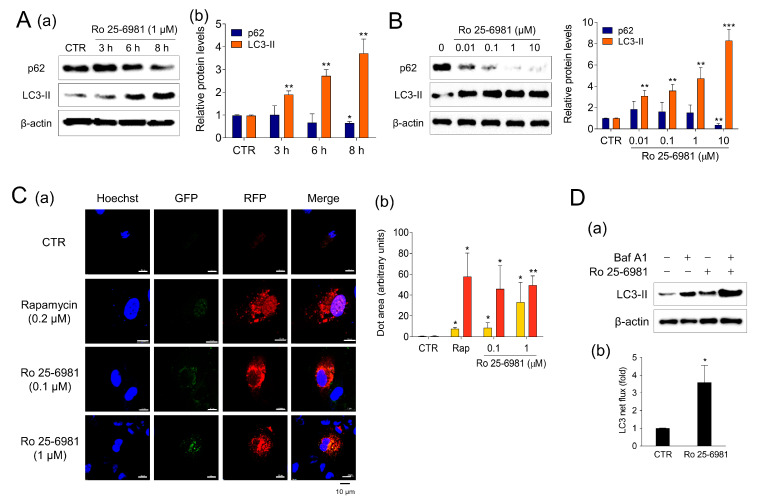
Ro 25-6981 activates autophagy in ARPE-19 cells. (**A**) ARPE-19 cells were treated with 1 μM Ro 25-6981, and the protein levels of LC3-II, p62, and β-actin were detected using western blotting (a). Images were quantified using the Image J software (b). (**B**) ARPE-19 cells were treated with various concentrations of Ro 25-6981 (0.01, 0.1, 1, and 10 μM) or vehicle (CTR) for 8 h. (**C**) Ro 25-6981 increases autophagic flux in ARPE-19 cells. Cells were transfected with a plasmid expressing RFP-GFP-LC3. Using confocal microscopy, nuclei stained with Hoechst 33342 (blue), GFP (green), and RFP (red puncta) were monitored. Cells treated with rapamycin (0.2 μM) were used as the positive control (a). Areas of autophagosomes (yellow puncta) and autolysosomes (red puncta) were quantified (b). (**D**) LC3-II levels in ARPE-19 cells treated with 10 nM bafilomycin A1 (Baf A1), 1 μM Ro 25-6981, or both for 8 h (a). Autophagic flux was determined by measuring the net LC3-II levels in the presence and absence of bafilomycin A1 (b). The results are presented as the mean ± S.D. (*n* = 3); * *p* < 0.05, ** *p* < 0.01, *** *p* < 0.001 vs. CTR.

Next, we investigated the mechanism by which Ro 25-6981 clears A2E. Autophagy, an intracellular recycling mechanism that degrades unnecessary or non-functional organelles, has been proposed to be involved in the degradation of A2E [25,26]. The effect of Ro 25-6981 on autophagy activity was evaluated by monitoring the expression of LC3 and p62/SQSTM1, which are well-known autophagy markers [27]. In ARPE-19 cells treated with 1 μM Ro 25-6981, the LC3-II levels increased and p62 levels decreased in a time- and concentration-dependent manner, showing a typical autophagy activation pattern (Figure 2A,B). Next, we observed the effect of Ro 25-6981 on autophagic flux using RFP-GFP-LC3 construct. Unlike red fluorescent protein (RFP), green fluorescent protein (GFP) is inactivated under acidic conditions, as in lysosomes, and is useful for monitoring autophagic flux [28,29]. Treatment with rapamycin, an inducer of autophagy, led to the formation of red puncta in ARPE-19 cells, indicating autolysosome formation. A similar result was observed in ARPE-19 cells treated with Ro 25-6981 (Figure 2C). Finally, after blocking autophagic flux with Baf A1, LC3-II formation further increased when ARPE-19 cells were treated with Ro 25-6981 (Figure 2D). These results suggest that Ro 25-6981 activated autophagy in ARPE-19 cells.

### 3.3. Ro 25-6981 Restores Autophagy in ARPE-19 Cells Damaged by A2E and BL

**Figure 3:**
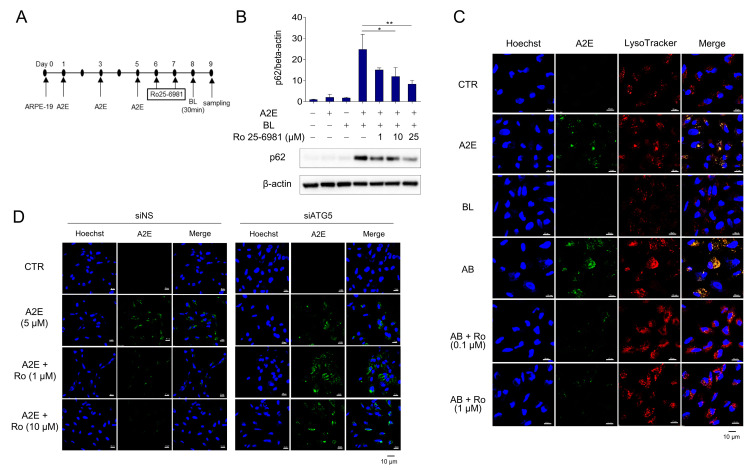
Ro 25-6981 restores autophagy in ARPE-19 cells damaged by A2E and BL. (**A**) Schematic schedule of AREP-19 cell treatment. (**B**) Effect of Ro 25-6981 treatment on autophagy in ARPE-19 cells with A2E (5 μM) accumulation and blue light (BL)-induced phototoxicity. ARPE-19 cells with A2E accumulation were exposed to BL (430 nm) and the expression of p62 was detected using western blotting. β-actin was used as a loading control. (**C**) Ro 25-6981 reduces A2E levels in BL-damaged ARPE-19 cells. A2E-laden ARPE-19 cells were treated with Ro 25-6981 (0.1 or 1 μM) and then exposed to BL for 30 min. The fluorescent signals were observed using fluorescence confocal microscopy. A2E (green); LysoTracker (red); Hoechst 33342 (blue); BL: blue light; AB: A2E + blue light. (**D**) Effect of Ro 25-6981 on A2E levels in ATG5-depleted ARPE-19 cells imaged using confocal microscopy. After 72 h of transfection with either siNS or siATG5, ARPE-19 cells were treated three times with 5 μM A2E at 48 h intervals, and then twice with Ro 25-6981 at 24 h intervals. The results are presented as the mean ± S.D. (*n* = 3); * *p* < 0.05, ** *p* < 0.01 vs. A2E + BL.

Autophagy is inhibited when A2E-treated RPE cells are exposed to BL [30]. Furthermore, the protein levels of p62 were further increased by A2E + BL treatment, suggesting enhanced inhibition of autophagy in ARPE-19 cells. Therefore, we investigated whether Ro 25-6981 could degrade A2E and restore the autophagic flux. The protein levels of p62 were significantly increased when ARPE-19 cells with accumulated A2E were exposed to BL, suggesting the blockade of autophagic flux. However, treatment with Ro 25-6981 reduced the enhancement in the levels of p62 caused by A2E + BL in a concentration-dependent manner (Figure 3A,B). Next, we tested whether A2E was cleared by Ro 25-6981 in the BL-induced phototoxicity model and found that Ro 25-6981 reduced A2E in a concentration-dependent manner (Figure 3C). To investigate whether A2E degradation by Ro 25-6981 is mediated by autophagy, we evaluated A2E clearance by Ro 25-6981 in ARPE-19 cells with impaired autophagic activity. In siNS-transfected control cells, A2E clearance was observed after treatment with Ro 25-6981. In contrast, the degree of A2E clearance upon Ro 25-6981 treatment was significantly reduced in ARPE-19 cells in which ATG5, one of the key factors for autophagy flux [31,32], was also reduced (Supplementary Figure S1 and Figure 3D). This finding suggests that Ro 25-6981 restores impaired autophagy flux induced by BL and A2E accumulation in ARPE-19 cells through the degradation of A2E.

### 3.4. Inhibition of NMDA Signaling Is Involved in Autophagy Activation and A2E Degradation in ARPE-19 Cells

**Figure 4:**
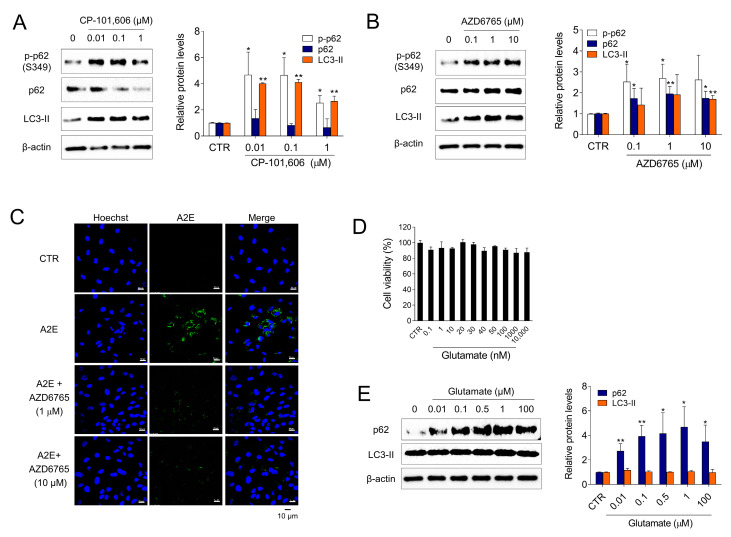
NMDA signaling is involved in autophagy and A2E degradation in ARPE-19 cells. (**A**,**B**) ARPE-19 cells were treated with CP-101,606 and AZD6765 for 8 h and the expression of LC3-II, p62, phospho-p62 (p-p62), and β-actin was detected using western blotting. (**C**) Effect of AZD6765 on A2E levels in ARPE-19 cells was examined using fluorescence confocal microscopy. ARPE-19 cells with accumulated A2E (5 μM) were treated with AZD6765 or a vehicle. The fluorescence signals were observed as Hoechst 33342 (blue) and A2E (green) in ARPE-19 cells. (**D**) Cell viability of glutamate-treated ARPE-19 cells. (**E**) Effect of 8 h glutamate treatment on autophagy in ARPE-19 cells. The expression levels of LC3-II, p62, and β-actin were detected using western blotting. The results are presented as the mean ± S.D. (*n* = 3); * *p* < 0.05, ** *p* < 0.01 vs. CTR.

To investigate whether Ro 25-6981 activated autophagy and degraded A2E through inhibition of the NMDA receptor pathway in ARPE-19 cells, the effect of other types of NMDA receptor antagonists (e.g., CP-101,606 and AZD6765) on the activation of autophagy was examined. Treatment with both CP-101,606 and AZD6765 increased the formation of LC3-II. Phosphorylation of p62 at Ser349 is an important event observed during autophagy [33,34]. The cargoes, together with phosphorylated p62 and KEAP1, are engulfed by autophagosomes and eventually degraded by autophagy. The levels of phospho-p62 (S349) was significantly increased upon treatment with CP-101,606 or AZD6765 (Figure 4A,B). Additionally, AZD6765 effectively cleared A2E from ARPE-19 cells (Figure 4C). In contrast, glutamate, an NMDA receptor agonist, significantly increased p62 and LC3-II levels at non-cytotoxic concentrations (Figure 4D,E), which implied that glutamate inhibits autophagy in ARPE-19 cells. Taken together, these results suggest that both the activation of autophagy and degradation of A2E by Ro 25-6981 occur through inhibition of the NMDA receptor pathway in ARPE-19 cells.

### 3.5. Inhibition of A2E Toxicity by Ro 25-6981 in ARPE-19 Cells

**Figure 5:**
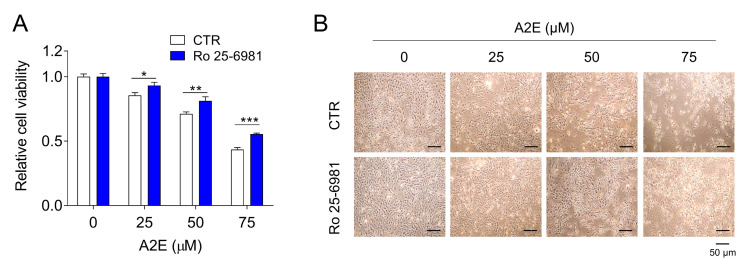
Effect of Ro 25-6981 on the viability of ARPE-19 cells damaged by A2E. (**A**) ARPE-19 cells grown in 96-well plates were treated with A2E and Ro 25-6981 (0.1 μM) for 24 h. After 24 h incubation, the cell viability was measured using cell counting. Data are presented as the mean ± S.D. (*n* = 3). * *p* < 0.05, ** *p* < 0.01, *** *p* < 0.001 vs. A2E-BDP. (**B**) Microscope images of ARPE-19 cells treated with A2E and Ro 25-6981.

Accumulation of A2E is known to induce cytotoxicity in ARPE-19 cells [23]. Based on this finding, we investigated whether Ro 25-6981 treatment affects the cytotoxicity caused by A2E in ARPE-19 cells. A decrease in the viability of ARPE-19 cells was observed upon A2E treatment in a dose-dependent manner. However, A2E-induced cytotoxicity was significantly inhibited upon treatment with Ro 25-6981 (Figure 5). These results support our previous finding that Ro 25-6981 clears A2E from ARPE-91 cells.

## 4. Discussion

Dry AMD, a retinal degenerative disease, is one of the most common diseases in people over the age of 50; however, there is no specific treatment for this disease [35]. Although the cause of dry AMD is unclear, age, gender, amount of light exposure, smoking, and inflammation are known to be associated factors [36,37]. The accumulation of lipofuscin in the retina is a characteristic feature of dry AMD [38,39]. A2E is a metabolite of vitamin A and a component of lipofuscin in the retina [40,41]. There is an association between the accumulation of A2E in RPE cells and apoptosis [42,43]. Photooxidation of A2E by BL, which is commonly encountered in daily life, promotes the death of RPE cells [44,45]. A previous study showed that photooxidation of A2E by blue light activates a wide variety of cellular physiological pathways in retinal pigment epithelial cells. These results suggest that blocking individual pathways is not effective in protecting retinal cells against blue light. Therefore, the most effective method to block the activated pathways is to remove lipofuscin, a phototoxic factor accumulated in cells in the process of aging. 

Attempts to inhibit A2E accumulation in retinal cells by using natural extracts have been reported [21,46,47]. However, since these extracts contain multiple physiologically active substances, it is difficult to ascertain the exact mechanism of A2E elimination. In addition, the individual components of the extracts can have opposite cellular biological actions; this poses another hurdle to understanding the mechanism of action. Therefore, we sought to identify a drug that eliminates A2E, using a compound library, with known mechanisms of action. Fluorescently labeled A2E-BDP was used to screen the compound library to identify a compound that cleared A2E without causing cytotoxicity. The NMDA receptor antagonist Ro 25-6981 was identified as a compound that significantly reduces A2E levels in a concentration-dependent manner in ARPE-19 cells. It has been reported that aging is accompanied by an increase in A2E accumulation and a decrease in autophagy activity in retinal cells [30,48]. Therefore, we focused on autophagy as a mechanism for eliminating A2E, a major photooxidative component of lipofuscin.

NMDA receptor antagonists act as antidepressants [49,50] and are used in Alzheimer’s treatment [51]. The connection between NMDA signaling and autophagy has been previously reported. For instance, MP1-MP2, an NMDA receptor antagonist, activates autophagy by regulating the Beclin 1 binding protein in glioblastoma multiforme [52]. Another NMDA receptor antagonist, memantine, has also been reported to activate autophagy [53,54]. In contrast, the blockade of NMDA receptors during stroke has been shown to inhibit autophagy [55]. Therefore, since contradictory results have been reported on the effect of NMDA receptor antagonists on autophagy activation, the role of NMDA signaling in autophagy in ARPE-19 cells remains unclear [56]. Our results show that NMDA receptor antagonists act as autophagy activators in human RPE cells and clear A2E. Ro 25-6981 reduced A2E accumulation in the lysosomes of ARPE-19 cells and enhanced autophagic flux at non-cytotoxic concentrations. Importantly, this phenomenon was not a selective function of Ro 25-6981 alone, but other types of NMDA antagonists also showed similar results. For example, other NMDA receptor antagonists, such as CP-101,606 and AZD6765, also increased the formation of LC3-II and effectively eliminated A2E in ARPE-19 cells. Furthermore, the NMDA receptor agonist glutamate showed opposite results, supporting the hypothesis that Ro 25-6981 activates autophagy and degrades A2E through inhibition of the NMDA receptor pathway in ARPE-19 cells. These results demonstrate a novel association between the NMDA signaling pathway and pathogenesis of retinal degeneration. Notably, recent studies reporting a correlation between AMD and Alzheimer’s disease support our findings [57,58].

## 5. Conclusions

Our results demonstrate that Ro 25-6981, an NR2B-selective NMDA receptor antagonist, effectively clears A2E, a component of ocular lipofuscin, in human RPE cells. A2E clearance by Ro 25-6981 is mediated by autophagy. Treatment with other NMDA receptor antagonists showed similar effects, including autophagy activation and A2E degradation in ARPE-19 cells. Taken together, our study suggests that NMDA receptor antagonists degrade lipofuscin via autophagy in human retinal pigment epithelial cells, and they could serve as useful tools for future research on retinal degenerative diseases.

## Data Availability

Not applicable.

## Supplementary Materials

The following supporting information can be downloaded at: https://www.mdpi.com/article/10.3390/medicina58081129/s1, Figure S1: Reduction in the levels of endogenous ATG5 by siRNA.
