# Supplementary material for: NMDA Receptor Antagonists Degrade Lipofuscin via Autophagy in Human Retinal Pigment Epithelial Cells
Source: Medicina (Kaunas). 2022 Aug 20;58(8):1129. doi: 10.3390/medicina58081129 (PMC9415004; doi:10.3390/medicina58081129)
Supplement: Supplementary file 1 [file medicina-58-01129-s001.zip › medicina-1813744-supplementary.pdf]

Supplementary Figure

Figure S1

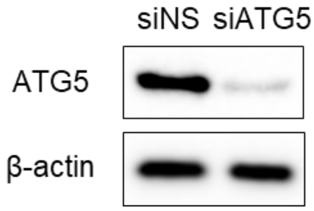

Supplementary Figure S1. Reduction in the level of endogenous ATG5 by siRNA. ARPE-19 cells were transiently transfected with siRNAs targeting *ATG5* mRNA (siATG5) or non-specific siRNA (siNS) for 72 h. Endogenous ATG5 protein levels in ARPE-19 cells were measured by western blotting.
